# Supplementary material for: Clinical, serological and epidemiological features of hepatitis A in León, Nicaragua
Source: PeerJ. 2021 Jun 21;9:e11516. doi: 10.7717/peerj.11516 (PMC8223896; doi:10.7717/peerj.11516)
Supplement: Supplemental Information 2 [file peerj-09-11516-s002.pdf]

Supplemental material

Clinical, serological and epidemiological features of hepatitis A in León, Nicaragua

Table S1. Characteristics of study population for acute hepatitis A cases (n=315) by age, 2
Table S2. Characteristics of study population for non-hepatitis A cases (n=242) by age 4

Table S1: Characteristics of study population for acute hepatitis A cases (n=315) by age, n (%)

Characteristic	Category	≤1 year (n=2)	1 - ≤5 years (n=70)	5 - ≤10 years (n=182)	14 - ≤18 years (n=46)	14 - ≤18 years (n=10)	>18 years (n=5)	Total (n=315)
Demographics								
Sex								
	female	2 (100.0)	28 (40.0)	102 (56.0)	20 (43.5)	3 (30.0)	2 (40.0)	157 (49.8)
	male	0 (0.0)	42 (60.0)	80 (44.0)	26 (56.5)	7 (70.0)	3 (60.0)	158 (50.2)
Socioeconomic								
Housing								
	no crowding	1 (50.0)	27 (38.6)	78 (42.9)	27 (58.7)	5 (50.0)	3 (60.0)	141 (44.8)
	crowding	1 (50.0)	43 (61.4)	104 (57.1)	19 (41.3)	5 (50.0)	2 (40.0)	174 (55.2)
Toilets								
	outside	0 (0.0)	38 (54.3)	91 (50.0)	23 (50.0)	3 (30.0)	2 (40.0)	157 (49.8)
	inside	2 (100.0)	32 (45.7)	91 (50.0)	23 (50.0)	7 (70.0)	3 (60.0)	158 (50.2)
Water								
	outside	0 (0.0)	14 (20.0)	33 (18.1)	9 (19.6)	1 (10.0)	0 (0.0)	57 (18.1)
	inside	2 (100.0)	56 (80.0)	149 (81.9)	37 (80.4)	9 (90.0)	5 (100.0)	258 (81.9)
Clinical features								
Jaundice								
	no	0 (0.0)	1 (1.4)	12 (6.6)	1 (2.2)	0 (0.0)	0 (0.0)	14 (4.4)
	yes	2 (100.0)	69 (98.6)	170 (93.4)	45 (97.8)	10 (100.0)	5 (100.0)	301 (95.6)
Pale stool								
	no	2 (100.0)	42 (60.0)	116 (63.7)	36 (78.3)	8 (80.0)	3 (60.0)	207 (65.7)
	yes	0 (0.0)	28 (40.0)	66 (36.3)	10 (21.7)	2 (20.0)	2 (40.0)	108 (34.3)
Dark Urine								
	no	1 (50.0)	27 (38.6)	74 (40.7)	14 (30.4)	3 (30.0)	1 (20.0)	120 (38.1)
	yes	1 (50.0)	43 (61.4)	108 (59.3)	32 (69.6)	7 (70.0)	4 (80.0)	195 (61.9)
Nausea								
	no	2 (100.0)	23 (32.9)	45 (24.7)	10 (21.7)	2 (20.0)	1 (20.0)	83 (26.3)
	yes	0 (0.0)	47 (67.1)	137 (75.3)	36 (78.3)	8 (80.0)	4 (80.0)	232 (73.7)
Vomiting								
	no	1 (50.0)	18 (25.7)	48 (26.4)	8 (17.4)	4 (40.0)	2 (40.0)	81 (25.7)
	yes	1 (50.0)	52 (74.3)	134 (73.6)	38 (82.6)	6 (60.0)	3 (60.0)	234 (74.3)
Fever								
	no	2 (100.0)	19 (27.1)	29 (15.9)	13 (28.3)	1 (10.0)	0 (0.0)	64 (20.3)
	yes	0 (0.0)	51 (72.9)	153 (84.1)	33 (71.7)	9 (90.0)	5 (100.0)	251 (79.7)
Anorexic								
	no	0 (0.0)	18 (25.7)	30 (16.5)	5 (10.9)	2 (20.0)	0 (0.0)	55 (17.5)
	yes	2 (100.0)	52 (74.3)	152 (83.5)	41 (89.1)	8 (80.0)	5 (100.0)	260 (82.5)

Characteristic	Category	≤1 year (n=2)	1 - ≤5 years (n=70)	5 - ≤10 years (n=182)	14 - ≤18 years (n=46)	14 - ≤18 years (n=10)	>18 years (n=5)	Total (n=315)
Malaise								
	no	1 (50.0)	14 (20.0)	24 (13.2)	6 (13.0)	1 (10.0)	0 (0.0)	46 (14.6)
	yes	1 (50.0)	56 (80.0)	158 (86.8)	40 (87.0)	9 (90.0)	5 (100.0)	269 (85.4)
Laboratory findings								
Liver enzymes GOT								
	elevated	2 (100.0)	61 (87.1)	164 (90.1)	42 (91.3)	8 (80.0)	4 (80.0)	281 (89.2)
	normal	0 (0.0)	9 (12.9)	18 (9.9)	4 (8.7)	2 (20.0)	1 (20.0)	34 (10.8)
Liver enzymes GTP								
	elevated	2 (100.0)	62 (88.6)	168 (92.3)	44 (95.7)	9 (90.0)	4 (80.0)	289 (91.7)
	normal	0 (0.0)	8 (11.4)	14 (7.7)	2 (4.3)	1 (10.0)	1 (20.0)	26 (8.3)
Total serum bilirubin								
	elevated	2 (100.0)	55 (78.6)	161 (88.5)	42 (91.3)	10 (100.0)	4 (80.0)	274 (87.0)
	normal	0 (0.0)	15 (21.4)	21 (11.5)	4 (8.7)	0 (0.0)	1 (20.0)	41 (13.0)

Table S2: Characteristics of study population for non-hepatitis A cases (n=242) by age, n (%)

Characteristic	Category	≤1 year (n=16)	1 - ≤5 years (n=47)	5 - ≤10 years (n=64)	14 - ≤18 years (n=32)	14 - ≤18 years (n=11)	>18 years (n=72)	Total (n=242)
Demographics								
Sex								
	female	9 (56.3)	22 (46.8)	30 (46.9)	14 (43.8)	3 (27.3)	42 (58.3)	120 (49.6)
	male	7 (43.8)	25 (53.2)	34 (53.1)	18 (56.3)	8 (72.7)	30 (41.7)	122 (50.4)
Socioeconomic								
Housing								
	no crowding	6 (37.5)	26 (55.3)	30 (46.9)	14 (43.8)	5 (45.5)	54 (75.0)	135 (55.8)
	crowding	10 (62.5)	21 (44.7)	34 (53.1)	18 (56.3)	6 (54.5)	18 (25.0)	107 (44.2)
Toilets								
	outside	6 (37.5)	23 (48.9)	34 (53.1)	9 (28.1)	4 (36.4)	23 (31.9)	99 (40.9)
	inside	10 (62.5)	24 (51.1)	30 (46.9)	23 (71.9)	7 (63.6)	49 (68.1)	143 (59.1)
Water								
	outside	6 (37.5)	11 (23.4)	14 (21.9)	3 (9.4)	1 (9.1)	8 (11.1)	43 (17.8)
	inside	10 (62.5)	36 (76.6)	50 (78.1)	29 (90.6)	10 (90.9)	64 (88.9)	199 (82.2)
Clinical features								
Jaundice								
	no	2 (12.5)	9 (19.1)	13 (20.3)	3 (9.4)	1 (9.1)	20 (27.8)	48 (19.8)
	yes	14 (87.5)	38 (80.9)	51 (79.7)	29 (90.6)	10 (90.9)	52 (72.2)	194 (80.2)
Pale stool								
	no	13 (81.3)	37 (78.7)	51 (79.7)	25 (78.1)	10 (90.9)	67 (93.1)	203 (83.9)
	yes	3 (18.8)	10 (21.3)	13 (20.3)	7 (21.9)	1 (9.1)	5 (6.9)	39 (16.1)
Dark Urine								
	no	9 (56.3)	32 (68.1)	39 (60.9)	20 (62.5)	7 (63.6)	55 (76.4)	162 (66.9)
	yes	7 (43.8)	15 (31.9)	25 (39.1)	12 (37.5)	4 (36.4)	17 (23.6)	80 (33.1)
Nausea								
	no	12 (75.0)	21 (44.7)	24 (37.5)	8 (25.0)	5 (45.5)	28 (38.9)	98 (40.5)
	yes	4 (25.0)	26 (55.3)	40 (62.5)	24 (75.0)	6 (54.5)	44 (61.1)	144 (59.5)
Vomiting								
	no	6 (37.5)	24 (51.1)	26 (40.6)	11 (34.4)	8 (72.7)	52 (72.2)	127 (52.5)
	yes	10 (62.5)	23 (48.9)	38 (59.4)	21 (65.6)	3 (27.3)	20 (27.8)	115 (47.5)
Fever								
	no	5 (31.3)	14 (29.8)	10 (15.6)	7 (21.9)	2 (18.2)	34 (47.2)	72 (29.8)
	yes	11 (68.8)	33 (70.2)	54 (84.4)	25 (78.1)	9 (81.8)	38 (52.8)	170 (70.2)
Anorexic								
	no	6 (37.5)	12 (25.5)	11 (17.2)	3 (9.4)	5 (45.5)	31 (43.1)	68 (28.1)
	yes	10 (62.5)	35 (74.5)	53 (82.8)	29 (90.6)	6 (54.5)	41 (56.9)	174 (71.9)

Characteristic	Category	≤1 year (n=16)	1 - ≤5 years (n=47)	5 - ≤10 years (n=64)	14 - ≤18 years (n=32)	14 - ≤18 years (n=11)	>18 years (n=72)	Total (n=242)
Malaise								
	no	6 (37.5)	5 (10.6)	9 (14.1)	3 (9.4)	3 (27.3)	23 (31.9)	49 (20.2)
	yes	10 (62.5)	42 (89.4)	55 (85.9)	29 (90.6)	8 (72.7)	49 (68.1)	193 (79.8)
Laboratory findings								
Liver enzymes GOT								
	elevated	10 (62.5)	26 (55.3)	28 (43.8)	15 (46.9)	4 (36.4)	25 (34.7)	108 (44.6)
	normal	6 (37.5)	21 (44.7)	36 (56.3)	17 (53.1)	7 (63.6)	47 (65.3)	134 (55.4)
Liver enzymes GTP								
	elevated	9 (56.3)	23 (48.9)	22 (34.4)	11 (34.4)	2 (18.2)	26 (36.1)	93 (38.4)
	normal	7 (43.8)	24 (51.1)	42 (65.6)	21 (65.6)	9 (81.8)	46 (63.9)	149 (61.6)
Total serum bilirubin								
	elevated	12 (75.0)	24 (51.1)	28 (43.8)	17 (53.1)	7 (63.6)	30 (41.7)	118 (48.8)
	normal	4 (25.0)	23 (48.9)	36 (56.3)	15 (46.9)	4 (36.4)	42 (58.3)	124 (51.2)
