# Supplementary material for: Clinical, serological and epidemiological features of hepatitis A in León, Nicaragua
Source: PeerJ. 2021 Jun 21;9:e11516. doi: 10.7717/peerj.11516 (PMC8223896; doi:10.7717/peerj.11516)
Supplement: Supplemental Information 3 [file peerj-09-11516-s003.docx]

***DEPARTAMENTO DE MICROBIOLOGIA Y PARASITOLOGIA***

***FACULTAD DE CIENCIAS MEDICAS UNAN –LEON.***

***Estudio de hepatitis Viral –Datos Clínicos***

***Fecha: _________***

***Ficha#____________ Puesto médico que refiere:________________***

*Nombre: _______________________________________________________________*

*Nombre del padre o tutor: _________________________________________________*

*Dirección: _____________________________________________________________*

*Teléfono:________________ Celular:__________________*

*Fecha de nacimiento: ____________________ Edad: __________*

*Procedencia:____________________________________________________________*

*Sexo: M:_________ F:___________*

*Ocupación: Si:_________ No:__________ Cual: _______________*

*Nivel académico: Analfabeto:_____Primaria:_____Secundara:_____Universitario.____*

***Manifestaciones clínicas***

*Fecha de inicio de síntomas:____________________________________*

*Ictericia: Si _______ No _______ Anorexia: Si______ No_______*

*Náuseas: Si _______ No _______ Vómitos: Si______ No_______*

*Fiebre: Si _______ No_______ Malestar general: Si______ No_______*

*Coluria: Si _______ No _______ Acololia: Si______ No_______*

*Recibió alguna transfusión sanguínea: Si: ________ No: ________*

*Hace menos de un año: ________________ Hace más de un año: _____________*

*Padeció de hepatitis anterior mente: Si: _________ No: ________*

***DEPARTAMENTO DE MICROBIOLOIA Y PARASITOLOGIA***

***FACULTAD DE CIENCIAS MEDICAS, UNAN –LEON***

***Estudio de Hepatitis Viral - Datos Epidemiológicos***

**Fecha:____________**

**Ficha:___________________ Puesto medico que refiere: __________________**

Nombre del participante: **____________________________________**

Nombre del Padre o tutor: **___________________________________**

**VISITA MEDICA: Día: ______ Mes: ______ Año: _______**

***Datos epidemiológicos***

No de habitantes en su casa:____________ No de cuartos: ________

**Hacinamiento: Si: _________ No: _________**

**Fuente de agua:**

Intradomiciliar: __________ Extradomiciliar: ________ Pozo: _______

**Disposición de excretas:**

Inodoro: ___________ Letrina: __________ Fecalismo: _________

(English translation on pages 3 & 4)

TRANSLATION:

**DEPARTMENT OF MICROBIOLOGY AND PARASITOLOGY**

**FACULTY OF MEDICAL SCIEN CES UNAN –LEON.**

**Viral hepatitis study – Clinical data**

Date: _________

Filing card#____________ Refering health center:________________

Name: _______________________________________________________________

Name of father or legal guardian: _________________________________________________

Address: _____________________________________________________________

Phone:________________ Mobile:__________________

Birthday: ____________________ Age: __________

Origina:____________________________________________________________

Sex: M:_________ F:___________

Occupation: Yes:_________ No:__________ Which: _______________

Educational level: Analphabet:_____Primary:_____Secundary:_____University:____

**Clinical manifestations**

Date of first symptoms:____________________________________

Icterus: Yes _______ No _______ Anorexia: Yes______ No_______

Nausea: Yesi _______ No _______ Vomiting: Yes______ No_______

Fever: Yesi _______ No_______ Malaise (general): Yes______ No_______

Dark urine: Yes _______ No _______ Pale stool: Yes______ No_______

Receipt of blood transfusion: Yes: ________ No: ________

Since less than 1 year: ________________ More than 1 year ago: _____________

History of prior hepatitis: Yes: _________ No: ________

**DEPARTMENT OF MICROBIOLOGY AND PARASITOLOGY**

**FACULTY OF MEDICAL SCIEN CES UNAN –LEON.**

**Viral hepatitis study – Epidemiological data**

Date:____________

Filing card #:___________________ Refering health center: __________________

Name of participant: ____________________________________

Name of father or legal guardian: ___________________________________

MEDICAL VISIT: Day: ______ Month: ______ Year: _______

Epidemiological data

No of inhabitants in your house:____________ No of rooms: ________

Crowded conditions: Yes: _________ No: _________

Water source:

Inside house: __________ Outside house: ________ Well: _______

Disposal of excreta:

Indoor: ___________ Latrine: __________ In the open: _________
