# Supplementary material for: Clinical, serological and epidemiological features of hepatitis A in León, Nicaragua
Source: PeerJ. 2021 Jun 21;9:e11516. doi: 10.7717/peerj.11516 (PMC8223896; doi:10.7717/peerj.11516)
Supplement: Supplemental Information 4 — BoletinInformativo sobre Hepatitis. Prevencin y Control de Enfermedades. Anlisisde Salud. Da Mundial contra la Hepatitis [Information bulletin on hepatitis.Prevention and Control of Diseases. Health Analysis. World Day against Hepatitis].Spanish (31 July 2013). [file peerj-09-11516-s004.pdf]

Día Mundial contra la Hepatitis

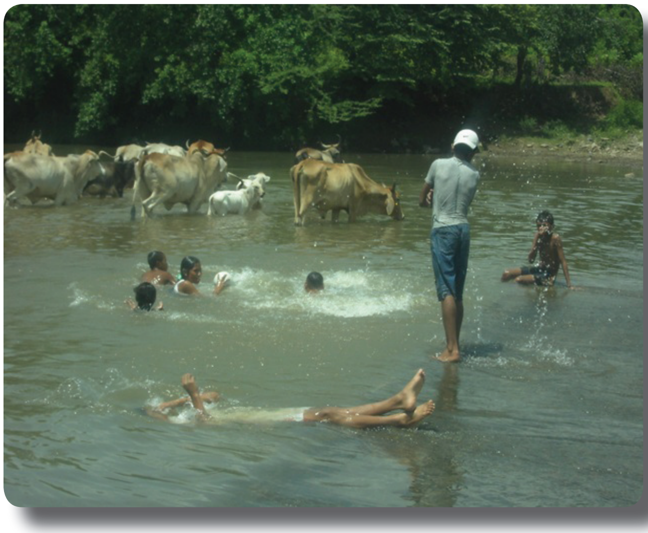

¿QUÉ ES LA HEPATITIS?

La hepatitis viral es una inflamación del hígado causada por uno de los cinco virus conocidos como A, B, C, D y E, que pueden causar enfermedad, discapacidad crónica y hasta la muerte. Las hepatitis A y E se transmiten principalmente a través de la ingesta de alimentos o agua contaminada. Los tipos B, C y D se transmiten básicamente a través de la sangre y fluidos corporales, lo que incluye a las prácticas sexuales no seguras.

DATOS Y CIFRAS

EL MUNDO

Actualmente, alrededor del 37% de la población mundial no tiene acceso a sistemas de saneamiento mejorados y el 11% no tiene acceso inmediato a fuentes de agua de bebida salubres. Esto significa que el agua insalubre y la falta de saneamiento son la norma para millones de personas (9).

Cada año se registran aproximadamente 1,4 millones de casos de hepatitis A en todo el mundo, aproximadamente 2000 millones de personas infectadas por el virus de hepatitis B y unos 150 millones de personas que padecen la infección crónica por Hepatitis C (11).

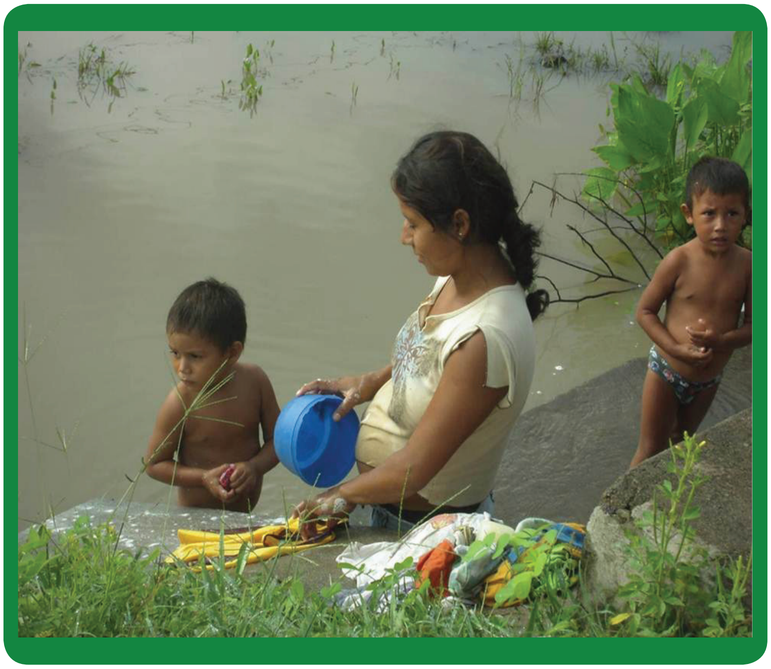

“Hay más infectados por hepatitis que por el VIH”

A nivel mundial, se estima que el 57% de los casos de cirrosis hepática y el 78% de los casos primarios de cáncer de hígado resultan de la infección con el virus de la hepatitis B o C (1).

LAS AMERICAS

En las Américas, la prevalencia de infección con el virus de la hepatitis B oscila de promedio de valores por debajo del 2% al 4% y; se estima que en América Latina y el Caribe entre 7 y 9 millones de adultos están infectados con el virus de la hepatitis C (1).

NICARAGUA

HEPATITIS "A"

El virus de la hepatitis A es una de las causas más frecuentes de infección de transmisión alimentaria principalmente por vía fecal-oral con consecuencias económicas y sociales graves en las comunidades. Los pacientes tardan semanas o meses en recuperarse y reanudar sus actividades laborales, escolares o cotidianas. No hay ningún tratamiento específico para la hepatitis A. La mejora del saneamiento, la inocuidad de los alimentos y la vacunación son las medidas más eficaces para combatir la hepatitis A. (3)

En nuestro país, la tasa de morbilidad por Hepatitis "A" presenta una tendencia al aumento, al pasar el numero de casos de 1088 (2005) a 1899 (2012), para una tasa de morbilidad actual de 3.69 x 10,000 habitantes (5).

Ilustración 1. Tendencia de la Morbilidad por Hepatitis "A" - Nicaragua, 2005-2012

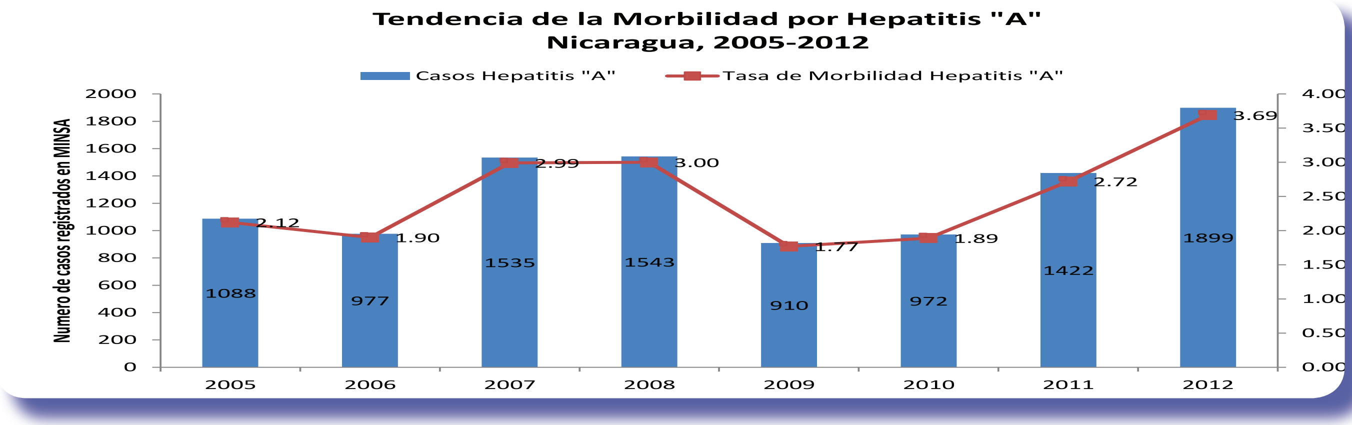

Fuente de Datos: Ministerio de Salud - Vigilancia Epidemiológica

Se pueden distinguir zonas geográficas de nivel elevado, intermedio o bajo de infección por VHA, siendo estas las siguientes:

HEPATITIS A																				
Lugares	2004		2005		2006		2007		2008		2009		2010		2011		2012		2005-2012	Peso porcentual
	Casos	Tasas	Casos	Tasas	Casos	Tasas	Casos	Tasas	Casos	Tasas	Casos	Tasas	Casos	Tasas	Casos	Tasas	Casos	Tasas	Total Casos	
Managua	299	2.37	388	3.07	382	3.02	521	4.13	351	2.78	233	1.84	238	1.88	204	1.62	301	2.38	2917	25.74
Jinotega	173	5.22	105	3.17	114	3.44	307	9.27	543	16.4	91	2.75	252	7.61	647	19.5	469	14.2	2701	23.84
Leon	45	1.26	67	1.88	88	2.47	131	3.68	98	2.75	37	1.04	23	0.65	79	2.22	309	8.69	877	7.74
Chinandega	82	2.16	78	2.06	70	1.85	141	3.72	79	2.08	93	2.45	108	2.85	88	2.32	81	2.14	820	7.24
Masaya	3	0.1	97	3.34	48	1.66	64	2.21	114	3.93	127	4.38	67	2.31	90	3.1	192	6.62	802	7.08
Matagalpa	9	0.17	42	0.81	51	0.98	52	1	109	2.1	77	1.49	82	1.58	70	1.35	81	1.56	573	5.06
Carazo	37	2.23	91	5.48	95	5.72	67	4.03	75	4.52	37	2.23	31	1.87	47	2.83	61	3.67	541	4.77
Rivas	33	2.11	63	4.03	40	2.56	112	7.17	38	2.43	35	2.24	42	2.69	6	0.38	46	2.94	415	3.66
Nueva Segovia	34	1.63	36	1.73	27	1.29	62	2.97	21	1.01	30	1.44	35	1.68	50	2.4	86	4.12	381	3.36
Esteli	62	3.08	33	1.64	14	0.69	6	0.3	53	2.63	5	0.25	18	0.89	64	3.18	84	4.17	339	2.99
Chontales	13	0.42	21	0.68	9	0.29	26	0.84	22	0.71	68	2.21	16	0.52	23	0.75	56	1.82	254	2.24
Madriz	14	1.06	8	0.6	4	0.3	26	1.96	15	1.13	50	3.77	37	2.79	20	1.51	79	5.96	253	2.23
R. A. Atlantico Sur	28	2.32	22	1.82	22	1.82	4	0.33	11	0.91	17	1.41	3	0.25	2	0.17	20	1.66	129	1.14
Granada	125	7.43					1	0.06									1	0.06	127	1.12
Boaco	26	1.73	11	0.73	1	0.07	6	0.4	9	0.6	9	0.6	7	0.46	1	0.07	23	1.53	93	0.82
Rio San Juan	2	0.21	23	2.41	8	0.84	8	0.84	2	0.21			9	0.94	21	2.2	7	0.73	80	0.71
Las Minas			1	0.06	2	0.12	1	0.06	1	0.06	1	0.06	3	0.18	9	0.54	2	0.12	20	0.18
Bilwi	1	0.08	2	0.15	2	0.15			2	0.15			1	0.08	1	0.08	1	0.08	10	0.09
Nicaragua	986	1.92	1088	2.12	977	1.90	1535	2.99	1543	3.00	910	1.77	972	1.89	1422	2.77	1899	3.69	11,332	100.00

Fuente de Datos: Ministerio de Salud - Vigilancia Epidemiológica

Zonas con altos niveles de infección (color rojo)

Son los SILAIS de Managua y Jinotega quienes registran el 49.58% de los casos del país (5), en ambos los factores comunes son la existencia de pilas con aguas estancadas donde se lavan manos para hacer necesidades fisiológicas, y en el caso particular de Jinotega, por el limitado acceso a saneamiento lo que conlleva a un fecalismo al aire libre (15% de su población total hace fecalismo al aire libre (6)).

Ilustración 2. Tendencia de los casos de morbilidad por Hepatitis “A” - SILAIS Managua y Jinotega, 2004-2012

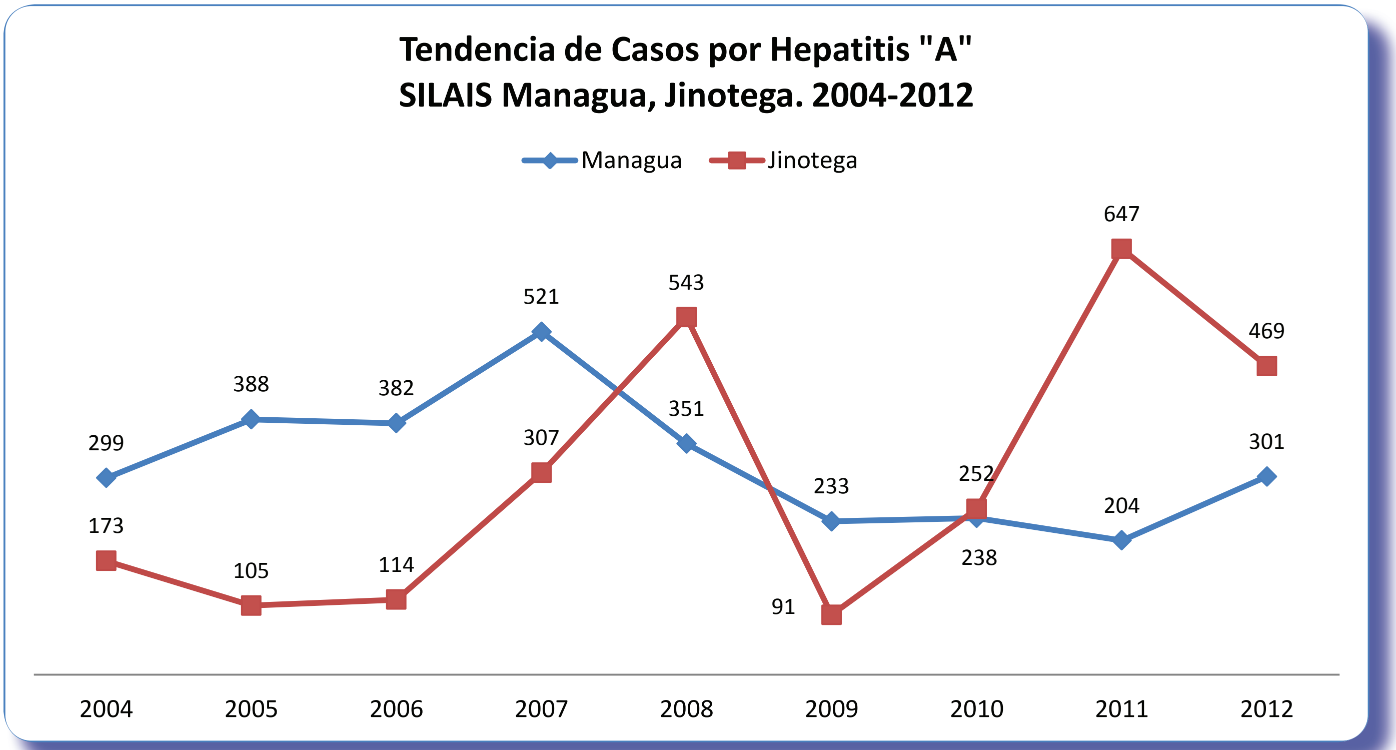

Fuente de Datos: Ministerio de Salud - Vigilancia Epidemiológica

En el caso del SILAIS Las Minas y Bilwi que aparece en zonas de baja infección debe considerarse el subregistro de notificación de las atenciones por este tipo de enfermedades.

HEPATITIS “B”

La hepatitis B es una infección hepática potencialmente mortal causada por el virus de la hepatitis B (VHB). Puede causar hepatopatía crónica y conlleva un alto riesgo de muerte por cirrosis y cáncer hepático. El VHB se transmite entre las personas por contacto directo de sangre a sangre o a través del semen o las secreciones vaginales de una persona infectada. Los modos de transmisión son los mismos que los del virus de la inmunodeficiencia humana (VIH), pero el VHB es entre 50 y 100 veces más infeccioso. A diferencia del VIH, el VHB puede sobrevivir fuera del organismo durante 7 días como mínimo, y en ese lapso todavía puede causar infección si penetra en el organismo de una persona no protegida por la vacuna.

No hay un tratamiento específico contra la hepatitis B aguda. Las medidas se centran en mantener el bienestar y el equilibrio nutricional, que incluye la reposición de los líquidos perdidos por los vómitos y la diarrea. (4)

En Nicaragua, la hepatitis B es considerada de baja endemicidad, esto respaldado por los resultados de la vigilancia epidemiológica, donde la prevalencia poblacional de VHB fue de 0,02%, con una cantidad de 12 casos en 2012 (5). A partir del año 2005, se observa una tendencia sostenida a la disminución en las tasas de notificación de Hepatitis B, especialmente marcada entre los años 2005 y 2009, para luego en el 2010 mostrar una estabilización (5).

Ilustración 3. Tendencia de la Morbilidad por Hepatitis "B"- Nicaragua, 2005-2012

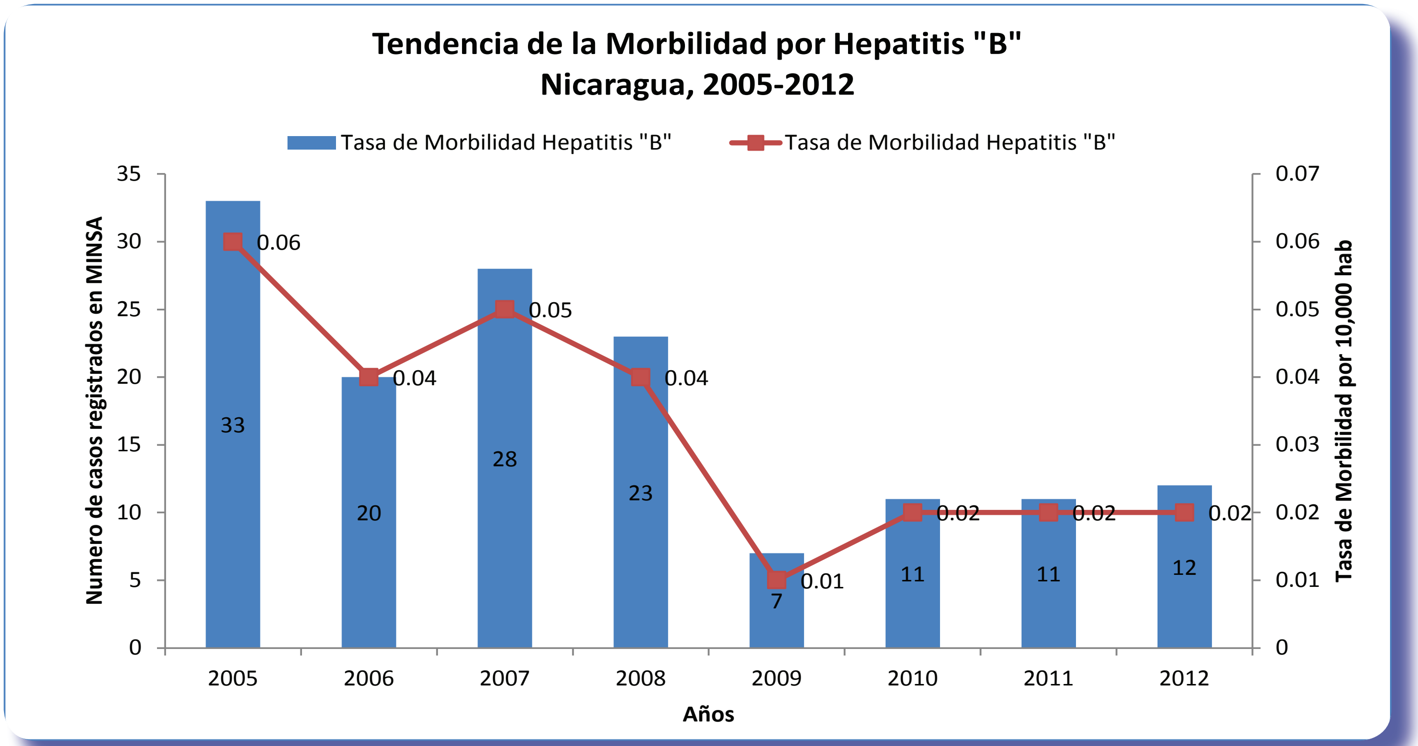

Fuente de Datos: Ministerio de Salud - Vigilancia Epidemiológica

Se pueden distinguir zonas geográficas de nivel elevado, intermedio o bajo de infección por VHB, siendo estas las siguientes:

Lugares	HEPATITIS B																			Peso porcentual
	2004		2005		2006		2007		2008		2009		2010		2011		2012		2005-2012	
	Casos	Tasas	Casos	Tasas	Casos	Tasas	Casos	Tasas	Casos	Tasas	Casos	Tasas	Casos	Tasas	Casos	Tasas	Casos	Tasas	Total Casos	
Managua	8	0.062	3	0.024	1	0.008	5	0.04	5	0.04	3	0.024	2	0.016	2	0.016	3	0.024	32	17.30
Boaco	3	0.20	13	0.86	7	0.465	2	0.13	2	0.13			2	0.13					29	15.68
Chontales	8	0.26	1	0.032			4	0.13	2	0.065	1	0.032	3	0.10	1	0.032			20	10.81
Bilwi	5	0.39	3	0.23	1	0.08	1	0.077	2	0.15			1	0.077	1	0.08	6	0.462	20	10.81
Madriz	4	0.302	1	0.08			5	0.377	1	0.08	1	0.08	1	0.08			1	0.075	14	7.57
Jinotega							6	0.15	6	0.15									12	6.49
Chinandega	2	0.05	2	0.05	3	0.08			2	0.053	1	0.026			1	0.03			11	5.95
Rivas	2	0.125	1	0.06	3	0.19	1	0.064							3	0.192			10	5.41
Rio San Juan			3	0.314	1	0.105	3	0.314							1	0.105			8	4.32
Las Minas	2	0.12			1	0.06	1	0.06	1	0.06							1	0.06	6	3.24
R. A. Atlantico Sur	2	0.17	1	0.08											2	0.166	1	0.083	6	3.24
Leon			1	0.03	1	0.025			1	0.03	1	0.03	1	0.03					5	2.70
Carazo	3	0.181	2	0.12															5	2.70
Esteli	1	0.05			1	0.05							1	0.05					3	1.62
Masaya			1	0.03	1	0.03			1	0.03									3	1.62
Matagalpa			1	0.019															1	0.54
Nueva Segovia																			0	0.00
Granada																			0	0.00
Nicaragua	40	0.08	33	0.06	20	0.04	28	0.05	23	0.04	7	0.01	11	0.02	11	0.02	12	0.02	185	100.00

Fuente de Datos: Ministerio de Salud - Vigilancia Epidemiológica

Zonas con altos niveles de infección (color rojo)

Son los SILAIS de Managua y Boaco quienes registran el 33% de los casos del país (5)

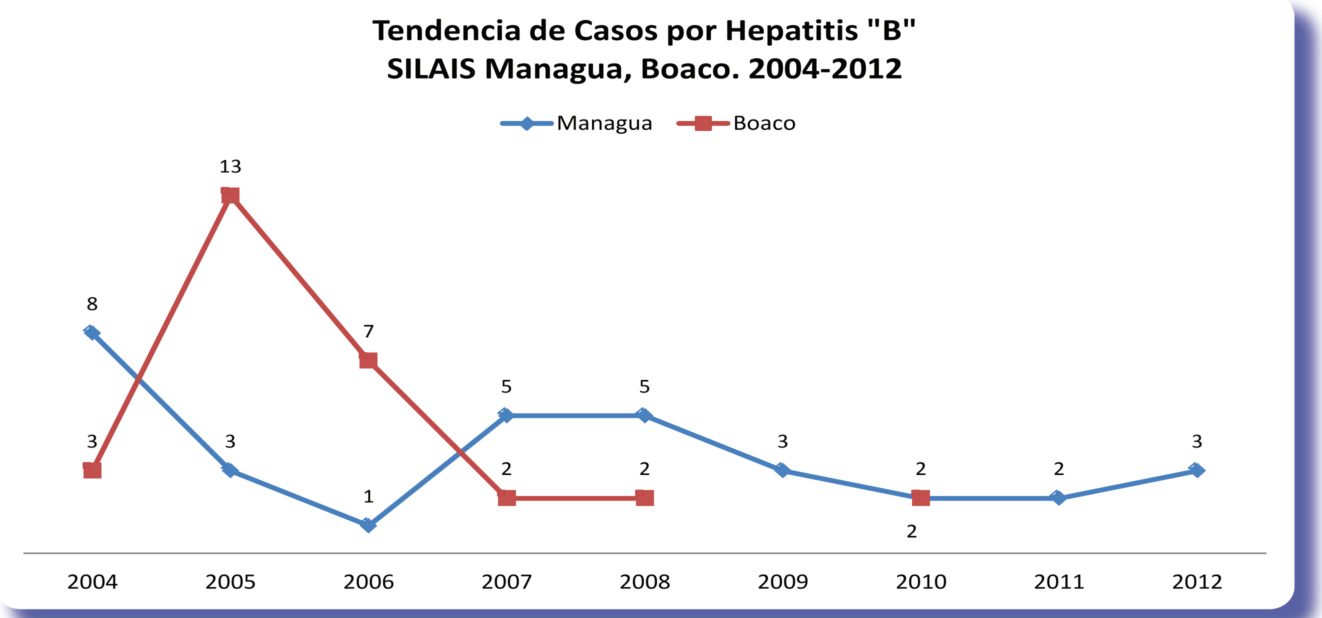

Fuente de Datos: Ministerio de Salud - Vigilancia Epidemiológica

VACUNA CONTRA HEPATITIS

En América Latina y el Caribe, la introducción de la vacuna contra la hepatitis B fue progresiva, aunque la mayoría de los países (26 países) introdujeron la vacuna entre 1997 y 2000; además, 13 países y territorios han incluido la dosis del recién nacido en sus programas de vacunación. Los países han introducido esta vacuna usando diferentes modalidades, siendo la vacuna pentavalente (DTP+Hib+Hep B) la combinación más usada (35 países y territorios). En cuanto a los niveles de cobertura de vacunación con tercera dosis de hepatitis B/pentavalente, en 2009, se encontraban por encima del 90% a nivel regional (12).

Ilustración 4. Mapa de cobertura de inmunización con 3ra dosis de Hepatitis B en Infantes - 2012

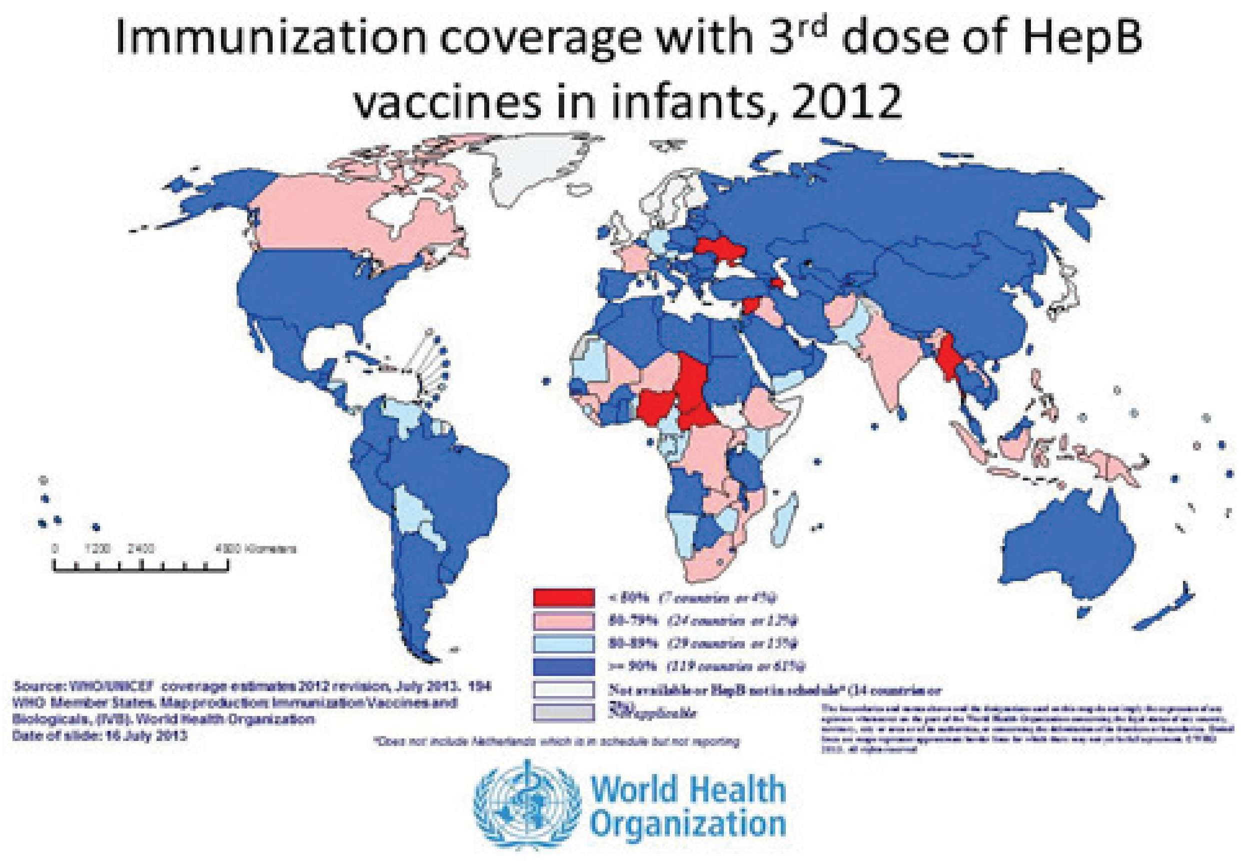

Fuente de Datos: Organización Mundial de la Salud. Immunization surveillance, assessment and monitoring / http://www.who.int/immunization_monitoring/diseases/hepatitis/en/index.html#

En Nicaragua, según el esquema de vacunación la vacuna pentavalente (DPT/HB, +Hib) la edad de inicio de la vacunación es a los dos meses de nacido, es del tipo intramuscular.

Los registros del Ministerio de Salud, evidencian coberturas de vacunación por este biológico en menores de un año por encima del 95% (8). Se calcula que la cobertura mundial con la vacuna contra el VHB es del 75% y en la actualidad está por debajo del objetivo mundial del 90% de cobertura (9).

Ilustración 5. Coberturas de vacunación por vacuna Pentavalente - Nicaragua

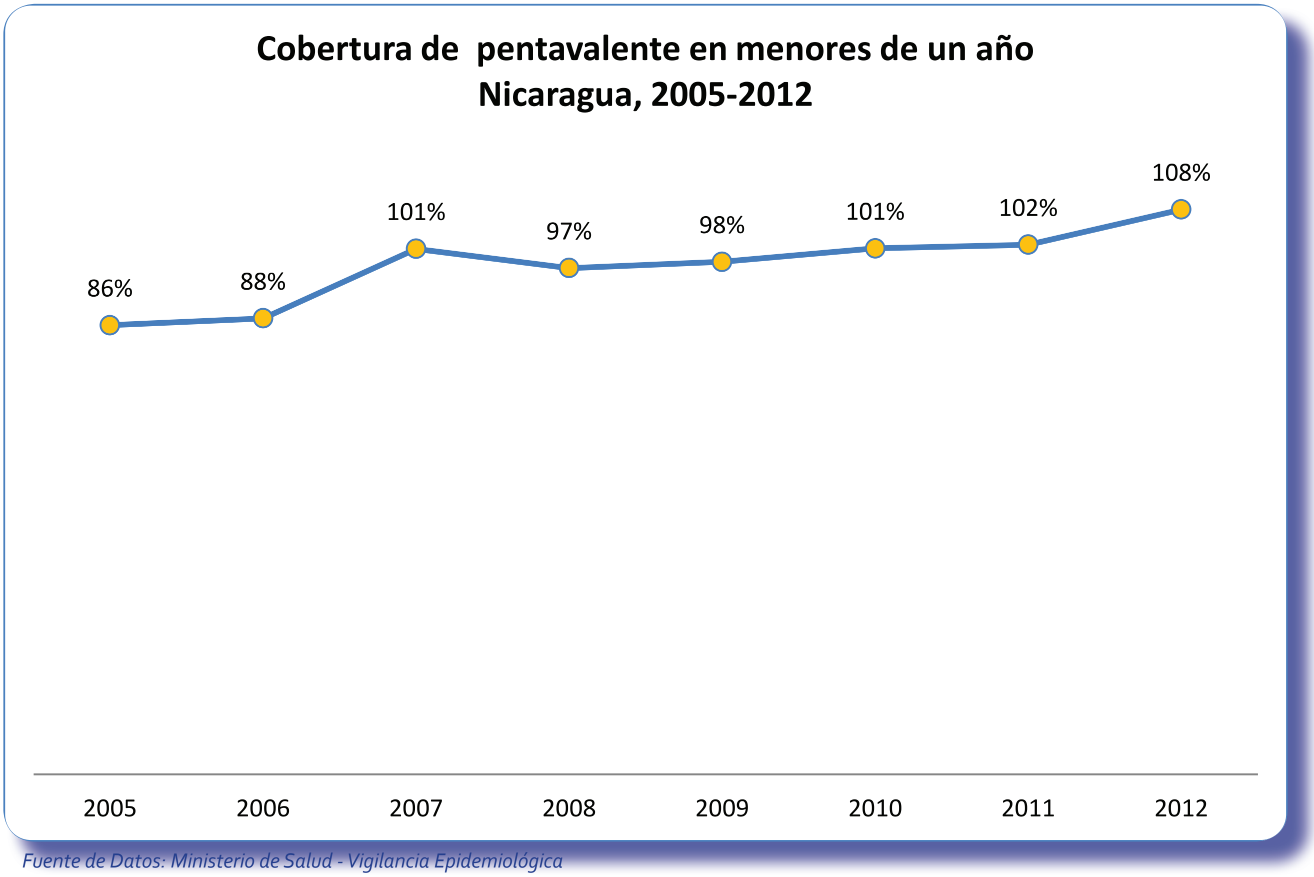

Fuente de Datos: Ministerio de Salud - Vigilancia Epidemiológica

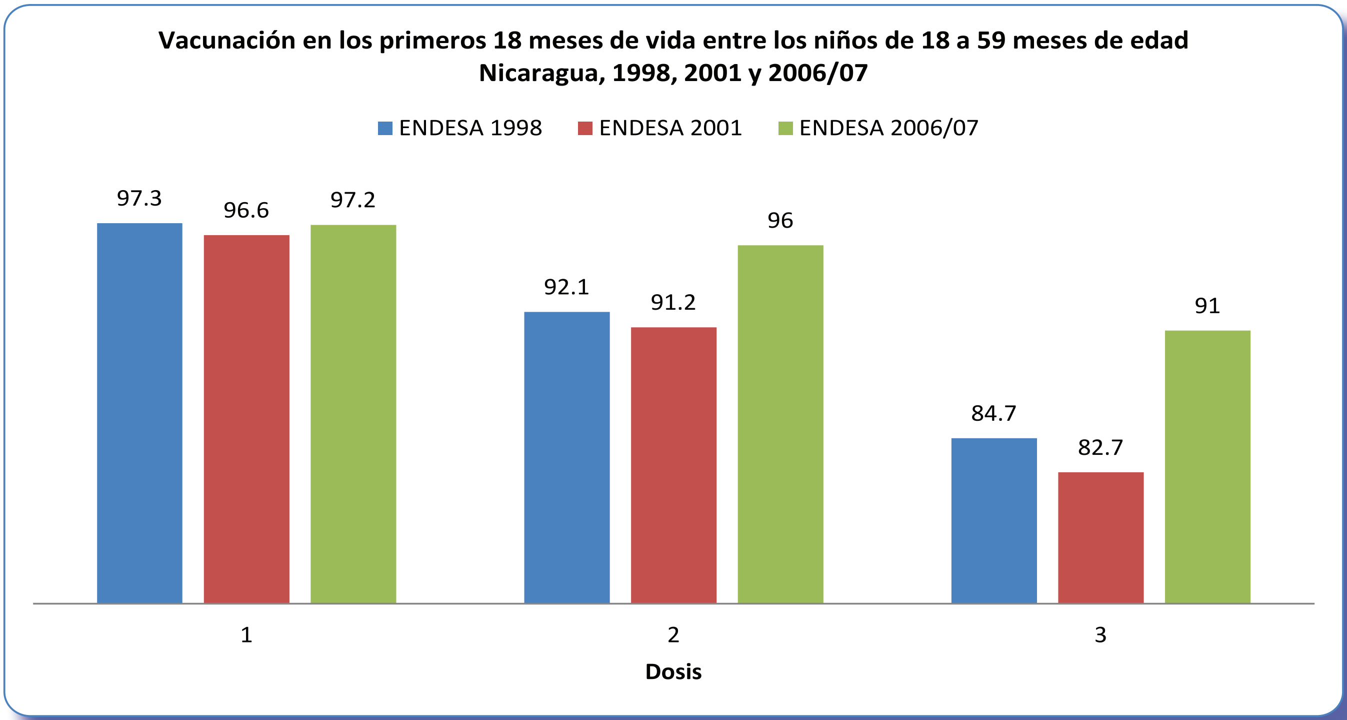

Fuente de Datos: INIDE - ENDESA 2006/07, 2011/12

Si bien las estadísticas de mortalidad en nuestro país aun presentan subregistros, se puede afirmar que son muy completas, habiéndose mejorado la asignación de la causa de muerte en las enfermedades en los certificados de defunción, sin embargo es necesario ampliar información para confirmar la etiología de esta enfermedad.

Tabla 1. Defunciones registradas por Hepatitis (B15 a B19) - Nicaragua

Causa de Defunción	1997	1998	1999	2000	2001	2002	2003	2004	2005	2006	2007	2008	2009	2010	2011	2012	Total
Hepatitis viral no especificada sin coma	14	13	7	14	9	10	7	10	14	9	4	9	13	11	5	5	154
Hepatitis aguda tipo B; sin agente delta y sin coma hepático	4	6	6	1	4	9	2	7	5	6	2	3		3	1	3	62
Hepatitis aguda tipo A; sin coma hepático	3	4	2		1	4	7	3	6		4		2	4	1	1	42
Hepatitis aguda tipo C		2	1			1		3		1		1	2	1		3	15
Hepatitis viral no especificada con coma	1		3						1				3		1		9
Hepatitis viral tipo C crónica									2						1	2	5
Hepatitis viral crónica; sin otra especificación				1							1		1	1			4
Hepatitis aguda tipo B; con agente delta (coinfección); con coma hepático										1						1	2
Hepatitis viral tipo B crónica; sin agente delta				2													2
Otras hepatitis virales agudas especificadas													1	1			2
Hepatitis aguda tipo A; con coma hepático					1												1
Hepatitis aguda tipo E																1	1
Total general	22	25	19	18	15	24	16	23	28	17	11	13	22	21	9	16	299

Fuente de Datos: Ministerio de Salud. Oficina Nacional de Estadisticas

MARCO NORMATIVOS - COMUNICACIÓN

Nacionales:

Constitución Política de Nicaragua

Arto. 59 Los nicaragüenses tienen derecho, por igual, a la salud. El Estado establecerá las condiciones básicas para su promoción, protección, recuperación y rehabilitación.

Arto. 105. Los servicios de educación, salud y seguridad social, son deberes indeclinables del Estado, que está obligado a prestarlos sin exclusiones, a mejorarlos y ampliarlos.

Ley 290. Ley de organización, competencia y procedimientos del poder ejecutivo.

Arto. 26. Funciones del Ministerio de Salud.

Ley No. 423. Ley general de Salud.

Internacionales:

La OMS está elaborando nueva detección de la hepatitis C, la atención y las pautas de tratamiento, que proporcionará recomendaciones sobre siete áreas clave, tales como el ensayo de enfoques, intervenciones conductuales (reducción del alcohol), la evaluación no invasiva de la fibrosis hepática, y la selección de combinaciones de fármacos contra la hepatitis C. (10)

RETOS

- Incrementar los esfuerzos y optimizar la coordinación de todos los sectores públicos/privados implicados, para desarrollar la estrategia nacional para la hepatitis viral, trabajando aun mas en el tratamiento de la hepatitis; fortaleciendo el programa de vigilancia de la hepatitis, específicamente en el seguimiento de la hepatitis B y C crónicas, que son responsables de enfermedades y muertes más severas.

- Fortalecer la detección, el diagnóstico y la terapia antiviral para la Hepatitis viral, como parte de las acciones preventivas del Modelo de Salud Familiar y Comunitario (MOSAFC), incluyéndose en las zonas de alta infección campañas de sensibilización y concientización sobre todos los tipos de hepatitis virales que ayuden a reducir la transmisión en la comunidad contra la hepatitis viral (9).

- ► De forma conjunta con grupos y asociaciones comunitarias desarrollar y poner en practica los lineamientos de los programas de prevención y control; siendo uno de los ejes trazadores las pruebas relativas a la detección y derivación en hepatitis B y la hepatitis C.

- ► Fortalecer la vigilancia epidemiológica y las estadísticas sanitarias con el fin de detectar los orígenes infecciosos, inmunitarios o tóxicos (abuso de alcohol, drogas o fármacos).

- ► Realizar de forma conjunta con el Ministerio de Salud de Nicaragua estudios epidemiológicos y de costo-efectividad sobre la introducción de la vacuna contra la hepatitis A para apoyar decisiones basadas en evidencia.

LOGROS

Desde 2009, OMS recomienda que se administre una dosis de una vacuna contra la hepatitis B en las 24 horas que siguen al nacimiento. En Nicaragua, se han logrado muchos avances en la prevención de la morbilidad y la mortalidad relacionadas con la hepatitis; la mayoría de las personas nacidas desde los años ochenta han estado protegidas en razón de la alta cobertura con las vacunas contra la hepatitis B. Recientemente se aplicaron 647, 651 dosis del biológico, vacunándose entre otros a 100,656 niños de 13 años de edad (7)

Nicaragua introdujo, además, vacunas como el rotavirus y neumococo, y realizó avances en función del saneamiento y acceso de la población al agua potable (13).

REFERENCIA BIBLIOGRÁFICA

1. Organización Panamericana de la Salud. Hepatitis en Las Américas. http://www.paho.org/par/index2.php?option=com_docman&task=doc_view&gid=429&Itemid=253

2. Organización Panamericana de la Salud. Nota de Prensa http://www.paho.org/col/index.php?option=com_content&view=article&id=1657:ops-llama-a-reforzar-lucha-contra-hepatitis-una-enfermedad-silenciosa-que-afecta-a-millones-de-personas-en-las-americas&catid=392&Itemid=487

3. Organización Mundial de la Salud. Nota Descriptiva Hepatitis “A” http://www.who.int/mediacentre/factsheets/fs328/es/index.html

4. Organización Mundial de la Salud. Nota Descriptiva Hepatitis “B” http://www.who.int/mediacentre/factsheets/fs204/es/

5. Ministerio de Salud de Nicaragua. Vigilancia Epidemiológica. Boletines Epidemiológicos de las Semana 52, casos y tasa acumuladas para los años 2005, 2006, 2007, 2008, 2009, 2010, 2011, 2012.

6. Instituto Nacional de Información de Desarrollo. Perfil de Departamento de Jinotega http://www.inide.gob.ni/censos2005/MONOGRAFIASD/JINOTEGA.pdf

7. El Pueblo Presidente. Nota de Prensa http://www.elpueblopresidente.com/salud/6347.html

8. Ministerio de Salud de Nicaragua. Oficina Nacional de Estadisticas. Indicadores Básicos de Salud 2005-2012.

9. Organización Mundial de la Salud. Prevención y control de las hepatitis virales: marco para la acción mundial
http://www.who.int/csr/disease/hepatitis/GHP_Framework_Es.pdf

10. 1Organización Mundial de la Salud. Nota de Prensa
http://www.europapress.es/salud/noticia-oms-pide-implicacion-gobiernos-contra-hepatitis-causa-14-millones-muertes-cada-ano-20130724142450.html

11. Organización Mundial de la Salud. Día Mundial contra la Hepatitis
http://www.who.int/csr/disease/hepatitis/world_hepatitis_day/es/index.html

12. Organización Panamericana de la Salud. Día Mundial contra la Hepatitis
http://www2.paho.org/HQ/index.php?option=com_docman&task=doc_view&gid=18241&Itemid=

13. Declaración de Dra Mirta Roses.
LA ORGANIZACIÓN PANAMERICANA DE LA SALUD, RECONOCIÓ EL LIDERAZGO DE NICA RAGUA EN LAS COBERTURAS DE VACUNACIÓN.
http://www.radiomaboas.cu/index.php/internacionales/america-latina/894-resalta-ops-logros-de-la-politica-de-salud-en-nicaragua

14. Constitución Política de Nicaragua
http://www.minsa.gob.ni/index.php?option=com_remository&Itemid=52&func=startdown&id=6830

15. Ley General de Salud
http://www.minsa.gob.ni/index.php?option=com_remository&Itemid=52&func=startdown&id=6832

16. Ley 290
http://www.minsa.gob.ni/index.php?option=com_remository&Itemid=52&func=startdown&id=6831

Elaborado por:
Ing. Julio Cajina - Oficial de Análisis de Salud
OPS-OMS Nicaragua
e-mail: cajinaj@nic.ops-oms.org

Aprobado por:
Dr. Wilmer Marquiño - Coordinador de Prevención y Control de Enfermedades
email: marquinw@nic.ops-oms.org

Diseño y diagramación
Area de Comunicación Social

Página Web http://new.paho.org/nic
